# Primary Localized Supraglottic Amyloidosis Presenting With Hoarseness: A Case Report

**DOI:** 10.7759/cureus.100761

**Published:** 2026-01-04

**Authors:** Duaa Alshammari, Khaled Al Abdulhadi, Essa Tawfeeq, Ahmad Hamad, Hussain Jarkhi

**Affiliations:** 1 Otolaryngology - Head and Neck Surgery, Ministry of Health, Kuwait, Jahra, KWT; 2 Otolaryngology - Head and Neck Surgery, Zain Hospital, Shwaikh, KWT; 3 Histopathology, Sabah Hospital, Shwaikh, KWT

**Keywords:** congo red staining, hoarseness, laryngeal amyloidosis, localized amyloidosis, supraglottic mass

## Abstract

Localized laryngeal amyloidosis is a rare condition, representing a small fraction of benign laryngeal lesions. Its clinical importance resides in its ability to mimic both benign and malignant tumors of the larynx, which often leads to a delayed diagnosis. The condition typically presents with hoarseness, although symptoms may range from mild vocal disturbances to significant airway compromise. Diagnosis requires histopathological confirmation with Congo red staining, as imaging and endoscopic findings alone are non-specific. Management is primarily surgical; functional preservation with endoscopic CO₂ laser excision is the preferred technique. Long-term monitoring and follow-up are required due to the frequent recurrence of the condition.

We report a case of isolated laryngeal amyloidosis in a 34-year-old male patient presenting with persistent hoarseness, highlighting diagnostic challenges, therapeutic considerations, and the need for vigilant surveillance.

## Introduction

Amyloidosis is a heterogeneous group of disorders that is characterized by the extracellular deposition of insoluble fibrils, which are derived from misfolded proteins. It can be classified as systemic, involving multiple organs, or localized, when confined to a single organ or site. Among its subtypes, localized laryngeal amyloidosis is an uncommon entity, comprising about 0.2-1.2% of benign laryngeal lesions [1-3]. The underlying pathophysiology involves abnormal folding and aggregation of immunoglobulin light chains, leading to localized amyloid deposition within the subepithelial tissue, most commonly affecting the false vocal cords and ventricles [1-4]. Despite its benign nature, this condition poses great difficulty in terms of diagnosis and treatment, as its clinical and radiological features may closely resemble those of malignant neoplasms [5-10].

Here, we describe the case of a 34-year-old male who presented with progressive hoarseness. His evaluation, which included advanced imaging, operative laryngoscopy, and biopsy, led to the diagnosis of localized supraglottic amyloidosis. A thorough systemic evaluation was negative, which reinforced the diagnosis of localized disease. This case highlights both the diagnostic pitfalls and the importance of multidisciplinary assessment in ensuring accurate diagnosis and appropriate management.

## Case presentation

A 34-year-old married male, a non-smoker and teetotaler with no significant medical or surgical history, presented to our otolaryngology clinic with a complaint of progressive hoarseness lasting more than two months. The onset of symptoms followed an episode of voice abuse. The patient initially presented to the emergency department in October 2024, where he was managed conservatively with medications aimed at relieving vocal strain and advised on voice rest. Despite this, his hoarseness not only persisted but also gradually worsened over two months, which led him to attend the ENT outpatient clinic.

The hoarseness was gradual in progression and described as a weak, monotone voice. Symptoms improved with rest and hydration but worsened significantly with vocal exertion. He denied associated throat pain, foreign body sensation, cough, throat clearing, hemoptysis, dysphagia, dyspnea, or systemic complaints such as fever or weight loss. He reported occasional heartburn and was on regular proton pump inhibitor therapy for reflux-related symptoms. Family history was not significant, and he worked in an occupation with minimal social interaction, with no known environmental or occupational exposures. He had no known allergies.

Given the persistence of symptoms, flexible fiberoptic laryngoscopy was performed, which revealed a supraglottic abnormality. This prompted further radiological evaluation. Contrast-enhanced CT of the neck revealed asymmetric fullness of the left piriform sinus and subtle mucosal thickening of the left vocal cord without evidence of a definite mass or cartilage invasion. MRI of the larynx and neck subsequently showed diffuse supraglottic edema with circumferential thickening involving the epiglottis and false vocal cords, which raised the possibility of an infiltrative process, but no destructive lesion was identified. The absence of a definable tumor mass made interpretation challenging and contributed to diagnostic uncertainty.

**Figure 1:**
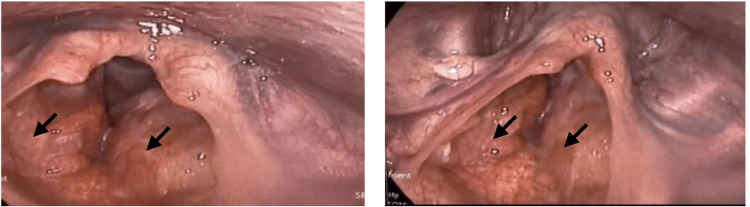
Flexible laryngoscopy: smooth, submucosal supraglottic mass (left-predominant) with intact mucosa

The flexible fiberoptic laryngoscopy revealed a supraglottic lesion appearing as a smooth, submucosal mass with intact overlying mucosa, predominantly on the left side. The lesion extended to the left piriform fossa and partially involved the left true vocal cord, with subtle restriction of mucosal wave, but cord mobility was preserved (Figure 1). The patient underwent direct microlaryngoscopy under general anesthesia. Intraoperatively, the mass was on the left supraglottis extending to the left piriform fossa, left vocal cord, anterior commissure, and the right supraglottis. The mass had a smooth surface, with intact mucosa and no evidence of ulceration or infiltration into adjacent cartilage. During the procedure, multiple biopsies were taken from the suspected regions. The right vocal cord, right piriform fossa, subglottic area, tongue base, and nasopharynx were all noted to be free. 

**Figure 2:**
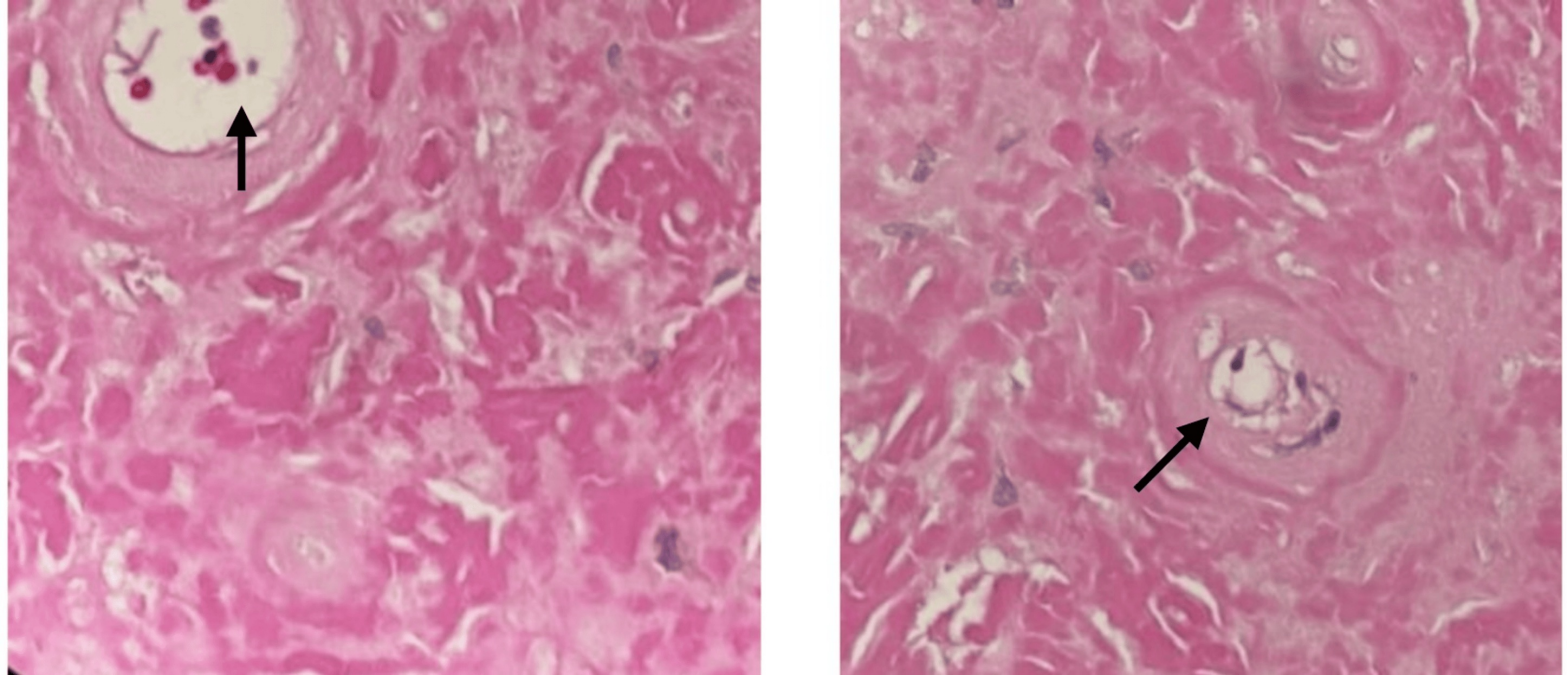
High power (x40) field of H&E-stained slide showing amorphous homogeneous extracellular deposits

**Figure 3:**
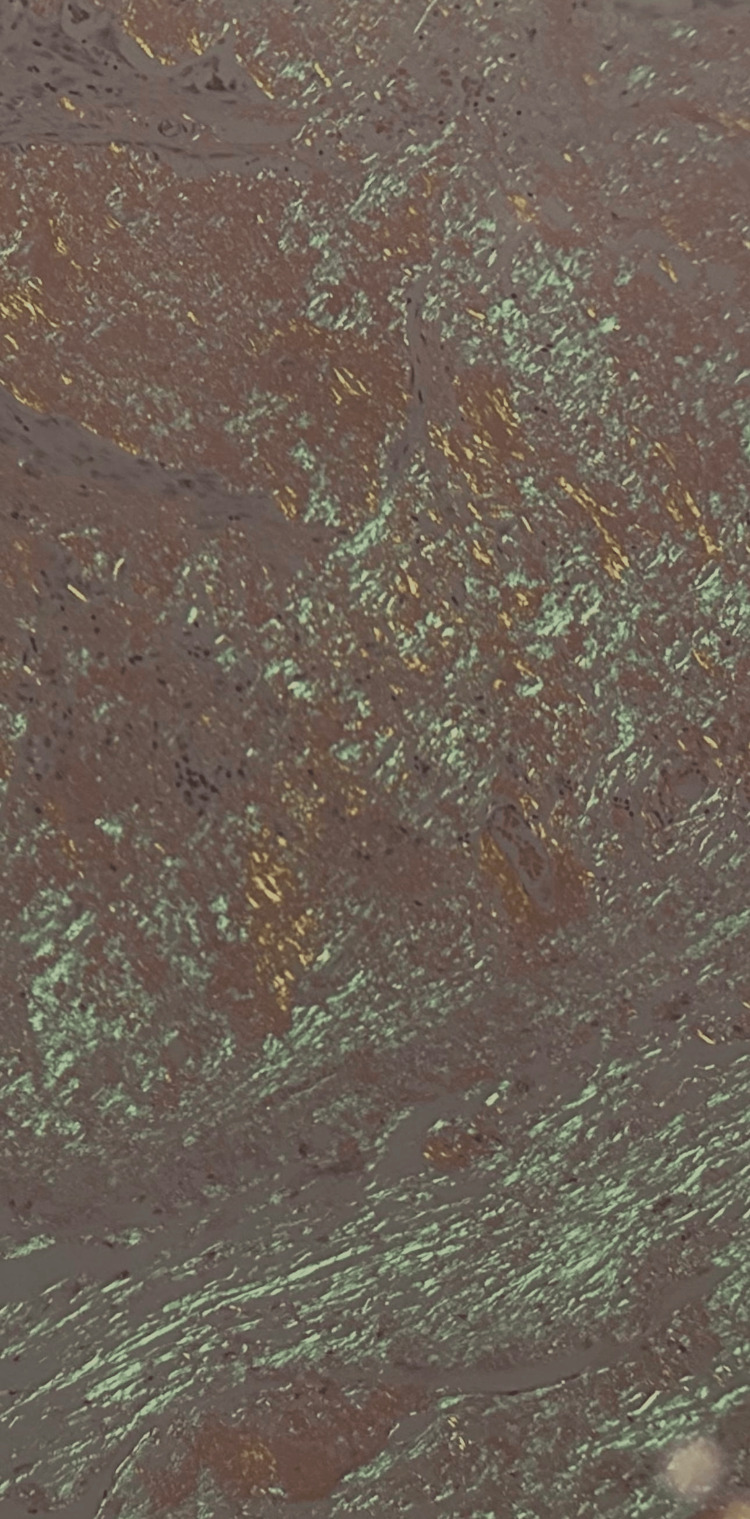
Histological section of Congo red stain, low power (x10) showing apple-green birefringence under polarization, a typical diagnostic feature of amyloidoma

Histopathological study demonstrated abundant eosinophilic, amorphous extracellular deposits beneath the mucosa (Figure 2). The tissue was stained with Congo red, which showed classic apple-green birefringence under polarized light, confirming the diagnosis of amyloidosis (Figure 3).

Following the pathological diagnosis, the patient underwent a systemic amyloidosis workup. Hematological investigations, including complete blood count, serum protein electrophoresis, and urine protein studies, were all within normal limits. Transthoracic echocardiography demonstrated normal cardiac structure and function, with no features of restrictive cardiomyopathy. Additionally, he underwent a CT scan of the chest, abdomen, and pelvis, which showed no evidence of amyloid infiltration in solid organs. Abdominal ultrasonography revealed normal liver and spleen with only mild nonspecific renal pelvicalyceal dilatation. Collectively, these findings excluded systemic amyloidosis and supported the diagnosis of primary localized supraglottic amyloidosis.

**Figure 4:**
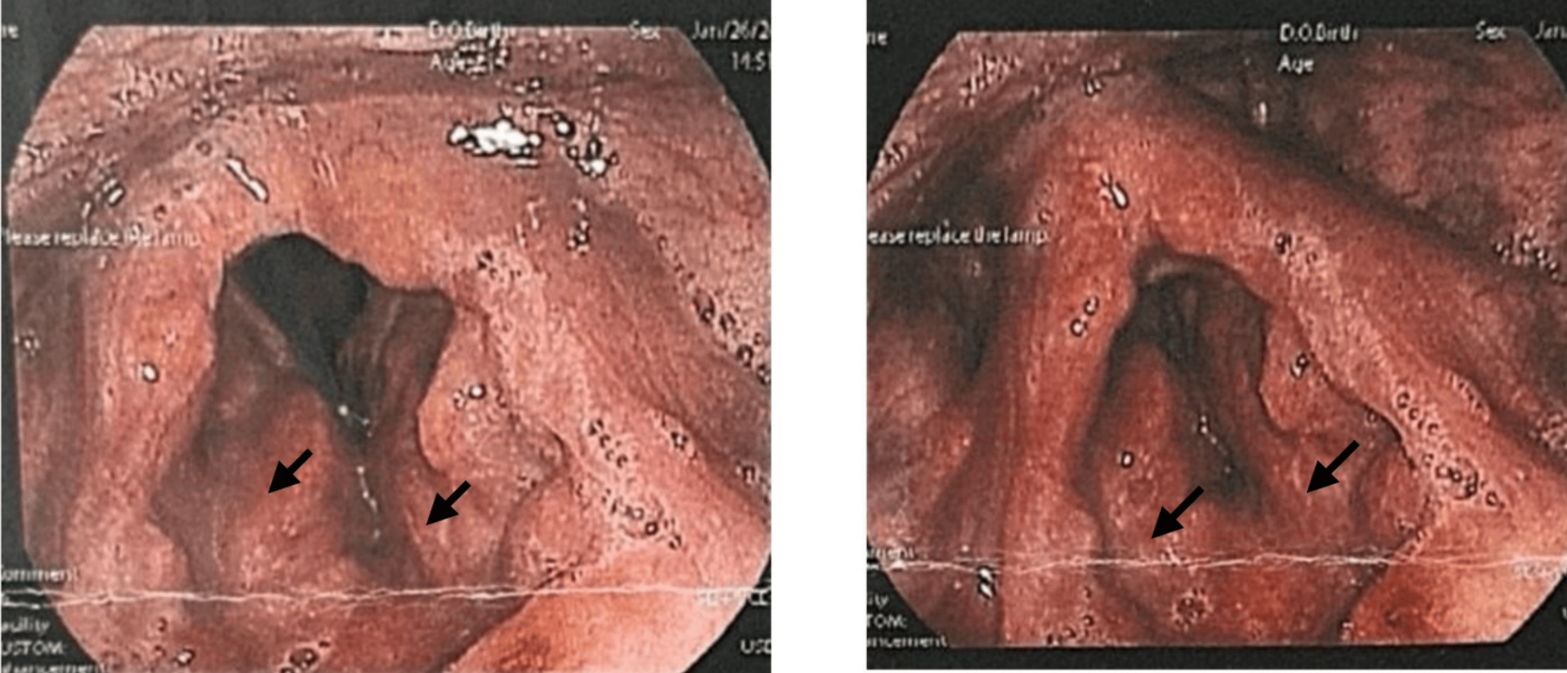
Surveillance laryngoscopy at the one-month follow-up in fully abducted position (A) and adduction (B), indicating stable supraglottic findings

**Figure 5:**
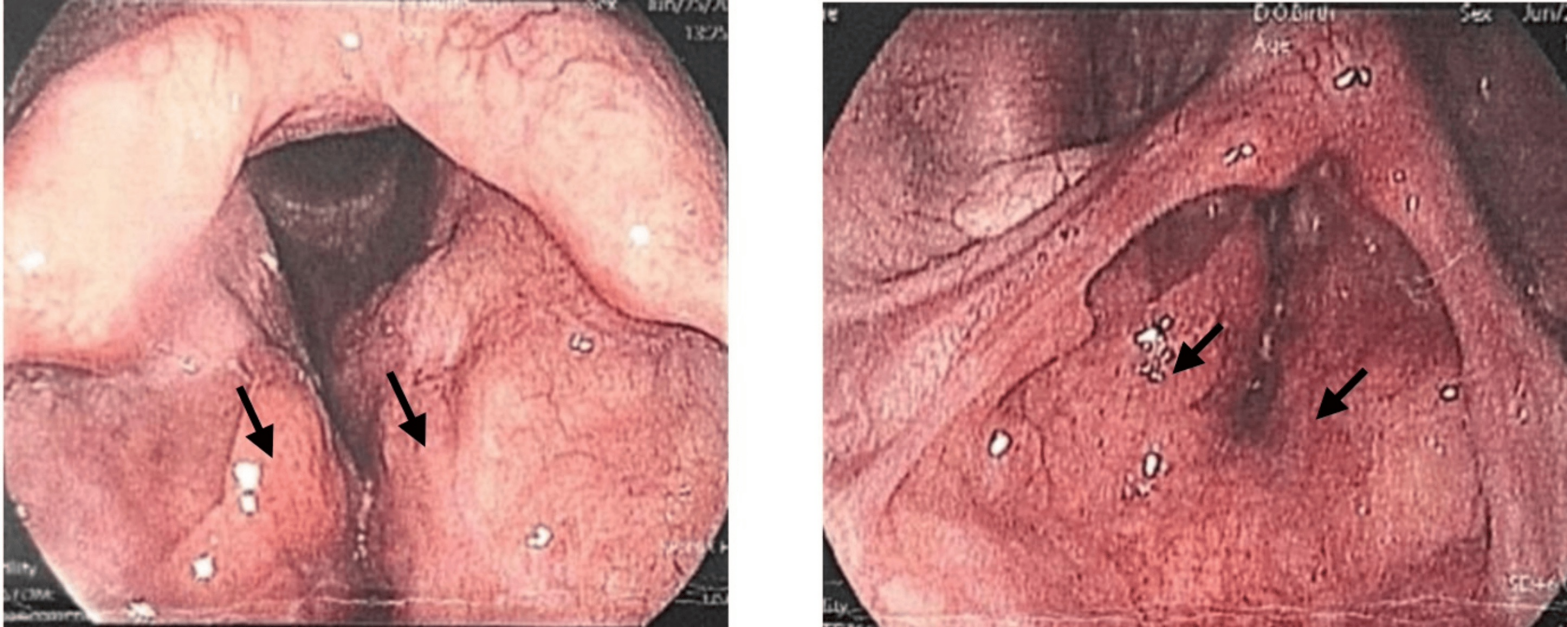
Surveillance laryngoscopy at the six-month follow-up in fully abducted position (A) and adduction (B), indicating stable supraglottic findings

The patient was placed on a structured follow-up schedule. Monthly endoscopic assessments were performed, which consistently demonstrated stable supraglottic findings without evidence of disease progression or airway compromise (Figures 4-5). Surgical intervention was deferred, as the lesion remained stable without evidence of airway compromise or symptom progression. The patient continued to be asymptomatic under regular follow-up with both the ENT and internal medicine teams, supporting the decision for conservative management and continued long-term surveillance given the risk of recurrence and multifocal disease.

## Discussion

Laryngeal amyloidosis, while histologically benign, poses a unique diagnostic and therapeutic challenge in otolaryngology. It is an uncommon condition, accounting for only 0.2-1.2% of benign laryngeal tumors, and has a distinct male predominance with peak incidence between the fourth and sixth decades of life [1-4]. It is most often localized in the supraglottic region, especially the false vocal cords and laryngeal ventricles, although the subglottis and true cords may also be involved [1-3]. The dominant symptom is progressive hoarseness, reflecting interference with vibratory function, but other symptoms, such as hemoptysis, dysphagia, dyspnea, or stridor in advanced cases, highlight its variable clinical spectrum [5-6]. Importantly, localized laryngeal amyloidosis rarely progresses to systemic disease but must always be considered in the differential diagnosis of submucosal laryngeal masses, given its ability to mimic carcinoma both clinically and radiologically [1,5,7].

The pathogenesis of laryngeal amyloidosis remains incompletely understood but is most consistently linked to local clonal plasma cells producing immunoglobulin light chains that misfold into insoluble β-pleated sheet fibrils [8-10]. These fibrils accumulate extracellularly in subepithelial tissues, resisting enzymatic degradation and provoking chronic inflammation. Histologically, deposits appear as homogeneous eosinophilic material surrounded by lymphocytic infiltration, plasma cells, and occasional giant cell reactions [2,8,10]. Immunohistochemistry often shows light chain restriction, supporting the theory of a localized clonal process. Interestingly, in rare cases, amyloid deposits have been linked to underlying mucosa-associated lymphoid tissue (MALT) lymphoma, underscoring the possibility of neoplastic lymphoplasmacytic activity as a pathogenic driver. Unlike systemic AL amyloidosis, which often progresses rapidly with poor prognosis, localized laryngeal amyloidosis usually follows a slow, indolent course, although rare aggressive cases with cartilage destruction and airway compromise have been reported [11].

Diagnosis is often overlooked due to the non-specific clinical presentation and resemblance to both benign lesions (such as polyps, cysts, or granulomas) and malignant tumors. If we were to consider the endoscopic view, amyloid typically presents as yellowish, submucosal nodules beneath intact mucosa, often multifocal [9-12]. Imaging plays an important adjunctive role: MRI is superior for delineating the soft tissue extent of lesions, while CT can demonstrate well-defined, non-erosive masses, helping exclude cartilage invasion or aggressive neoplasia [8,12,13]. However, radiology alone is insufficient for diagnosis, as both benign and malignant lesions may appear similar, which makes histopathological studies an essential key to diagnosis [5-7]. Congo red staining under polarized light remains the gold standard, producing the diagnostic apple-green birefringence [1-14]. Adjunctive staining, such as crystal violet, has been used to improve sensitivity, especially for small or interstitial deposits that are easily missed [10-15]. Advanced molecular methods, including mass spectrometry, provide definitive amyloid typing and are increasingly recommended in atypical or recurrent cases [14].

Because systemic amyloidosis dramatically changes prognosis, it is essential to first rule out generalized disease. A standard work-up usually includes serum and urine immunofixation, measurement of serum free light chains, and echocardiography, with bone marrow biopsy or abdominal fat pad aspiration performed when necessary [16]. In recent years, additional tools have been introduced to improve diagnostic accuracy; for example, potassium permanganate sensitivity testing, which helps distinguish AA from AL amyloid, and serum amyloid P (SAP) scintigraphy, which can confirm or exclude systemic deposits [6-7]. Together, these investigations not only reduce the risk of misclassification but also guide clinicians toward the most appropriate management strategy and provide patients with more accurate prognosis counseling.

Surgical excision remains the mainstay of therapy, with endoscopic CO₂ laser microlaryngoscopy regarded as the gold standard due to its precision, hemostatic capability, and minimal collateral injury [1,6,12,17,18]. The laser’s hydrophilic nature makes it particularly effective in ablating amyloid deposits [17]. Cold instruments may still be used in some cases, but laser excision is preferred because of the greater control and minimal scarring. Despite successful excision, recurrence is common, not due to incomplete resection but to the multifocal and infiltrative nature of amyloid deposition [4]. In fact, a large case series of 103 patients found that nearly 30% developed local progression and one-third already had multifocal disease at presentation [18]. Consequently, radical procedures like partial laryngectomy are not recommended, as the prevailing option is to perform conservative, function-preserving surgeries and be prepared for multiple operations [4,18].

Alternative non-surgical therapies remain limited in efficacy. Corticosteroids, melphalan, colchicine, and other systemic agents have shown inconsistent results, with little impact on recurrence rates [11,17,18]. Radiotherapy has been reported as a salvage option in recurrent or inoperable cases, though evidence is limited and its routine use is not recommended [18]. Treatment should be individualized: while symptomatic patients benefit from surgical intervention, asymptomatic or stable cases may be managed conservatively with careful monitoring [5]. This approach balances the need to preserve voice and airway function against the reality of recurrence and multifocal disease.

Prognosis for localized laryngeal amyloidosis is generally favorable, with survival often exceeding a decade [17]. However, long-term follow-up is critical. Surveillance for at least 5-7 years is widely recommended, as new foci may develop within the larynx or tracheobronchial tree after initial excision [8,12,18]. Follow-up should include regular endoscopic examination of the entire airway, not just the primary site, to identify multifocal recurrence early. The rare but significant association with extranodal marginal zone B-cell MALT lymphoma further highlights the need for ongoing hematologic evaluation in cases with atypical histological or radiologic features [19]. Emerging diagnostic tools, including SAP scans, Congo red fluorescence, and molecular amyloid typing, along with potential novel therapies targeting serum amyloid P and fibril clearance, illustrate the dynamic and evolving landscape of amyloidosis management [6,7,15,20].

In summary, laryngeal amyloidosis, though rare, is a clinically relevant condition with the potential to mimic malignancy and significantly impair laryngeal function. It arises from localized plasma cell-derived light chain deposition and is best managed with conservative CO₂ laser excision, supported by long-term surveillance. While prognosis is excellent in localized cases, recurrence and multifocality remain the rule rather than the exception, necessitating close follow-up. Associations with hematologic malignancies, advances in diagnostic techniques, and emerging systemic therapies broaden the academic and clinical importance of this disease. Ultimately, our understanding of laryngeal amyloidosis continues to improve, and each reported case contributes to refining strategies for diagnosis, management, and long-term outcomes [1,20].

## Conclusions

Laryngeal amyloidosis, though histologically benign, remains a clinically significant entity due to its rarity, nonspecific presentation, and potential to mimic malignancy. Histopathological confirmation with Congo red staining is critical for diagnosis, while systemic evaluation is mandatory to exclude generalized disease. Conservative surgical management, most commonly with endoscopic CO₂ laser excision, provides symptomatic relief, but recurrence is frequent, underscoring the need for vigilant, long-term surveillance.

Our case of a young male presenting with progressive hoarseness highlights the importance of considering amyloidosis in the differential diagnosis of submucosal laryngeal lesions, even when imaging is inconclusive. It reinforces that biopsy remains the diagnostic key, and that a multidisciplinary approach involving otolaryngology, anesthesiology, pathology, and internal medicine is crucial to ensure optimal outcomes.
